# Obstructed Hemivagina with Ipsilateral Renal Agenesis: A Challenging Case Report and a Management Flow Chart

**DOI:** 10.3390/jcm12237227

**Published:** 2023-11-22

**Authors:** Ewelina Malanowska-Jarema, Andrzej Starczewski, Yana Osnytska, Mariola Krzyścin, Elżbieta Sowińska-Przepiera, Matteo Balzarro, Emanuele Rubilotta

**Affiliations:** 1 Department of Gynecology, Endocrinology and Gynecologic Oncology, Pomeranian Medical University, 70-204 Szczecin, Poland; e_malanowska1@tlen.pl (E.M.-J.); andrzejstarcz@tlen.pl (A.S.); 2 Department of Endocrinology, Metabolic and Internal Diseases, Pomeranian Medical University, 70-204 Szczecin, Poland; krzyscin@o2.pl (M.K.); elzbieta.sowinska.przepiera@pum.edu.pl (E.S.-P.); 3 Department of Urology, Azienda Ospedaliera Universitaria Integrata Verona, Piazzale Stefani 1, 37100 Verona, Italy; matteo.balzarro@aovr.veneto.it (M.B.); emanuele.rubilotta@aovr.veneto.it (E.R.)

**Keywords:** obstructed vagina, OHVIRA syndrome, recurrent urinary tract infections (rUTI), congenital anomalies of kidney and urinary tract (CAKUT), renal agenesis, laparoscopic surgery in female adolescents

## Abstract

We present here a case of complex uterine anomaly—obstructed hemivagina with ipsilateral renal agenesis (OHVIRA), also known as Herlyn-Werner-Wunderlich syndrome in a 13-year-old girl with a history of recurrent urinary tract infections (rUTI). In the emergency room, a trans-abdominal sonography revealed an ovarian cyst and renal agenesis, without any suspicion of vaginal obstruction. This led to a delay in the diagnosis of this uncommon anomaly. Finally, MRI findings confirmed the presence of OHVIRA syndrome. As the congenital anomalies of the kidney and urinary tract (CAKUT) are present in almost one third of cases associated with genital malformations, urologists should carefully screen patients with rUTI. The patient underwent simultaneous laparoscopy and vaginoscopy, which was in our opinion the most appropriate therapeutic decision. In this article, we are also going to discuss the role of laparoscopy in the management of OHVIRA syndrome, as well as other surgical techniques described in the literature.

## 1. Introduction

Congenital anomalies of the kidney and urinary tract (CAKUT) are present in almost one third of cases associated with genital malformations [1,2]. The prevalence of CAKUT has a large range in the pediatric population (3.5% to 43%), and this cluster of disorders represents the most frequent cause of chronic kidney disease (CKD) in children [3]. Indeed, these disorders may predispose to recurrent urinary tract infections (rUTI), resulting in kidney impairment [4]. Uterus didelphys with obstructed hemivagina (OHVIRA) may cause acute abdominal pain, rUTI, and this condition may be associated with CAKUT [5]. Therefore, OHVIRA should be considered in young females with these symptoms and CAKUT.

Currently, the management of OHVIRA is still debated. Although many ideas for OHVIRA management have been suggested, delayed diagnosis is still an obvious problem. In this article, we present a problem-solving flow chart and other methods that could help professionals in their practice. Several operative techniques for OHVIRA management have been reported, such as puncture of the vaginal wall, single-stage vaginoplasty, mini-laparotomy and even unilateral hysterectomy [6]. However, evidence regarding the most effective therapeutic option is scant. Moreover, some authors have also questioned the diagnostic role of laparoscopy, which is still under debate [7].

We report a case of a young woman with OHVIRA associated with CAKUT, the condition of rUTI and pain. This case emphasizes that uterus didelphys with obstructed hemivagina (OHVIRA) should be considered in the differential diagnosis of adolescent girls presenting with acute abdominal pain, rUTI and CAKUT. Furthermore, we reviewed the most used surgical procedures for these conditions.

## 2. Case Report

We report the case of a 13-year-old girl who presented with acute abdominal pain and a UTI. Hematuria was not present. The patient had a history of rUTIs that occurred six times in the last year, with urine cultures positive for more than 10^5^ colony-forming units (CFU)/mL of *Escherichia coli* (*E. coli*). The young woman complained of rUTI despite courses of antibiotics that had been administered (Cefuroxime 2 × 1500 mg iv.).

The patient attained menarche 6 months previously, reporting of back and lower abdominal pain and UTI; it was of 1 week’s duration. She had regular menstrual cycles, every 30 days, lasting 3 to 4 days.

**Figure 1:**
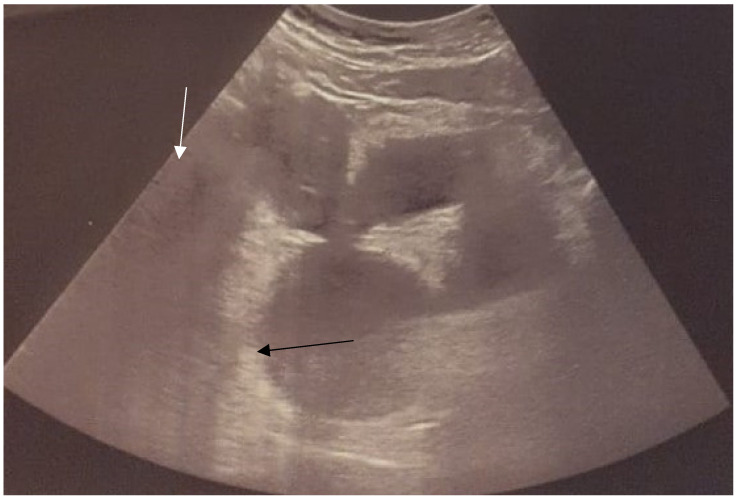
Ultrasound from extern hospital, when the diagnosis of hematocolpos was mistaken as an ovarian cyst (black arrow). Double uterus was not recognized (white arrow).

Abdominal ultrasonography (US) (Figure 1) revealed the right renal agenesis and suspicion of a right-ovarian cyst. Due to elevated tumor markers (Ca-125/ROMA Index), the patient was referred to laparotomy. Urgent urological consultation excluded any urological cause and indicated a gynecological background of the symptoms. Sudden vaginal bleeding led to expanding investigations, and the patient was admitted by tertiary referral to hospital.

Particular image examination was performed at the Department of Gynecology, Endocrinology and Gynecologic Oncology of University hospital.

**Figure 2:**
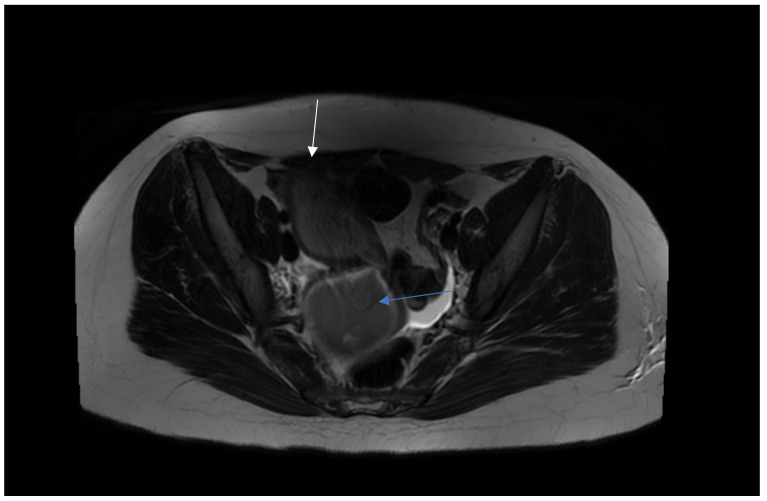
MRI presents hematocolpos (blue arrow) and hydrometrocolpos in right uterus (white arrow).

**Figure 3:**
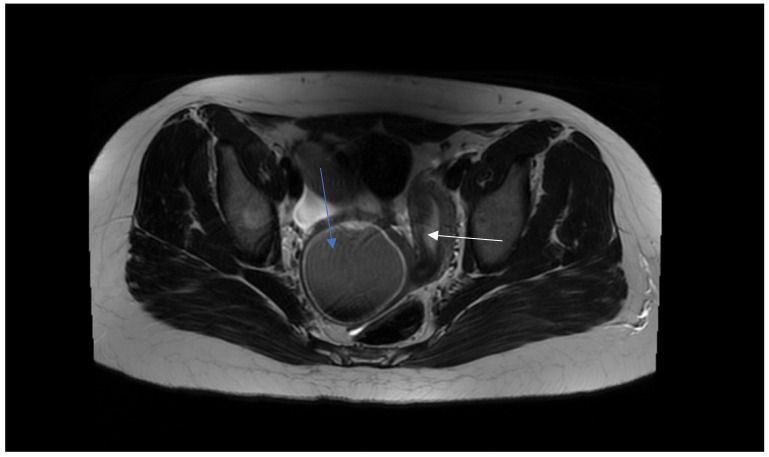
MRI presents hematocolpos (blue arrow) and a left healthy uterus (white arrow).

On admission, because of the patient’s severe pain, the examination was conducted under general anesthesia and the dorsal lithotomy position with a virgin-size speculum revealed a single normal vagina and one cervix. Bulging of the vaginal wall on the right side was also found. Unfortunately, the attempt to puncture the thick vaginal wall to relieve patient’s symptoms failed in providing drainage. MRI (Figure 2 and Figure 3) provided the correct diagnosis of both CAKUT and OHVIRA syndrome, and was crucial for the patient’s management and short- and long-term follow up. A rare congenital urogenital anomaly, uterus didelphys with obstructed hemivagina and ipsilateral renal agenesis (OHVIRA), known in the literature as Herlyn-Werner-Wunderlich (HWW) syndrome, was the final diagnosis.

According to the literature, we considered hemivaginal septal resection and anastomosis between the obstructed hemivagina and the normal vagina. However, escalating abdominal symptoms prompted us to perform laparoscopy simultaneously. 

### 2.1. Family History

The medical, family, and psychosocial history of the patient, including genetic information, revealed no history of renal abnormalities.

### 2.2. Preoperative Management

Pre-operative management included psychological counselling of the patient and her family regarding treatment options, with detailed description of a planned surgical approach. Before completion of the whole examination, the patient was given an oral combined estrogen–progestin pill to block recurrent uterine blood flow, which could intensify abdominal symptoms. 

### 2.3. Surgical Technique

**Figure 4:**
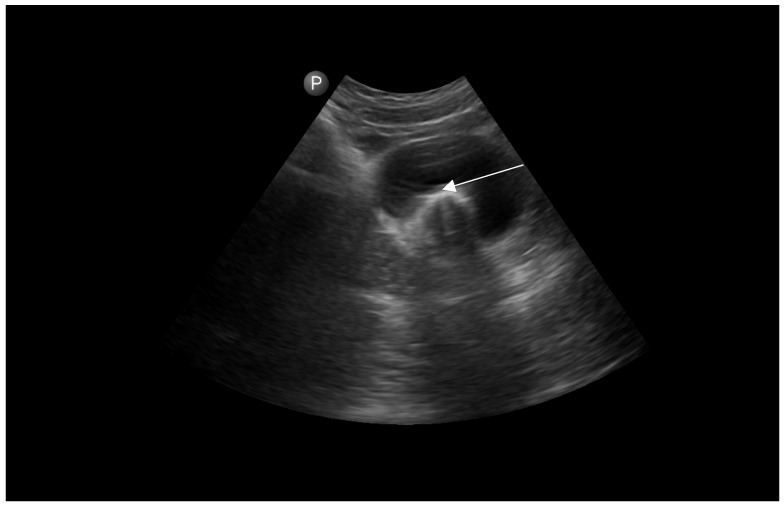
Intraoperative abdominal sonography and puncture of hematocolpos (white arrow). P is polish equivalent to R-right in english site of the ultrasound screen.

**Figure 5:**
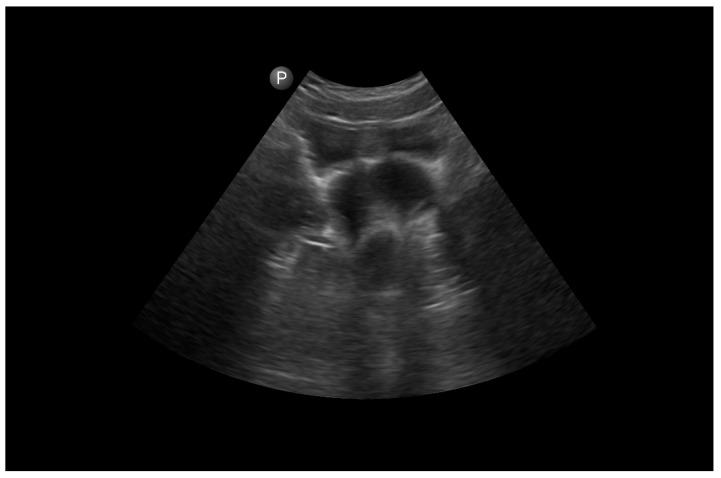
Intraoperative abdominal sonography and drainage of hematocolpos. P is polish equivalent to R-right in english site of the ultrasound screen.

**Figure 6:**
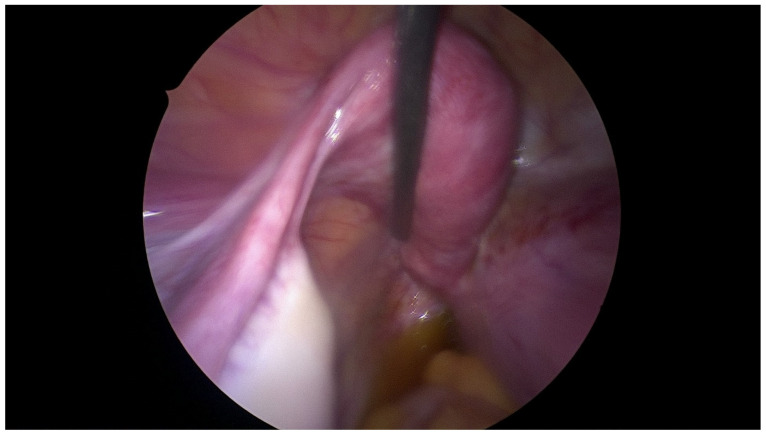
Left healthy uterus in laparoscopic view.

**Figure 7:**
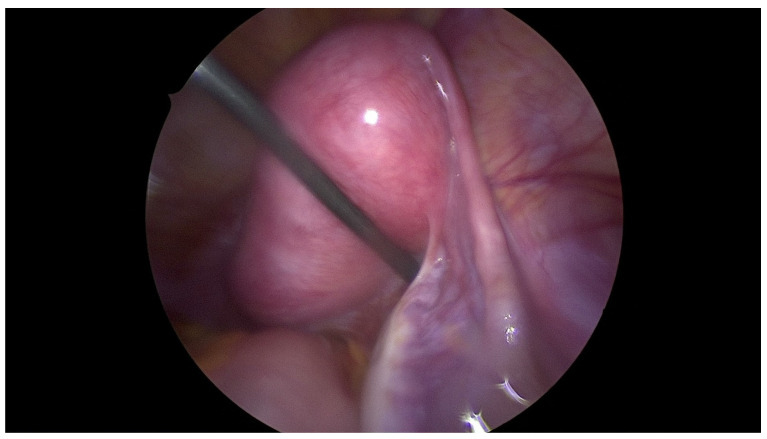
Right uterus with hematometra in laparoscopic view.

**Figure 8:**
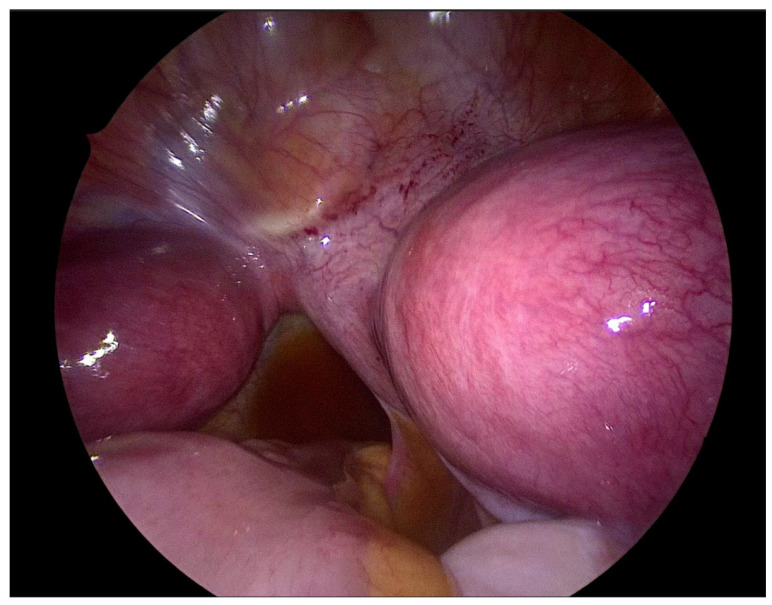
Double uterus. Right uterus with hematometra.

**Figure 9:**
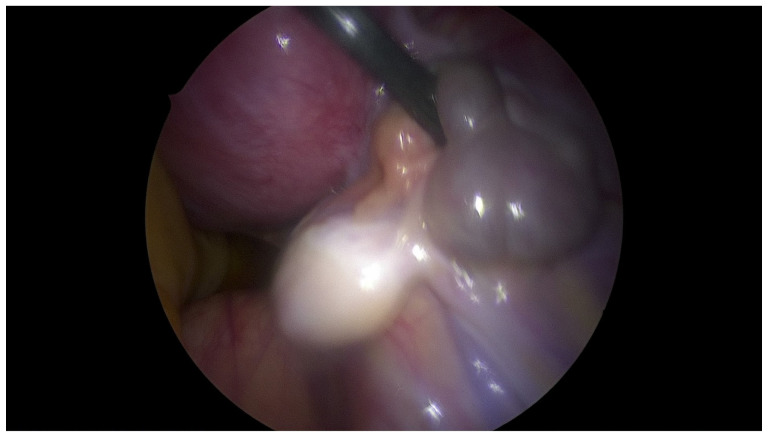
Hematosalpinx of right fallopian tube.

**Figure 10:**
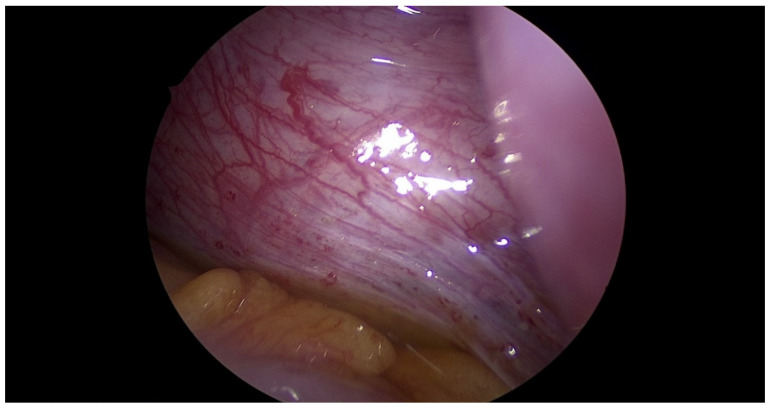
Active endometriosis of peritoneum.

The patient underwent combined laparoscopy and vaginoscopy. There was clinical evidence to suspect adnexal torsion and hematosalpinx, hence the laparoscopy was performed simultaneously. Vaginoscopy was completed, and with the help of a resectoscope, a vertical incision was created, the vaginal septum was excised and the vaginal opening was extended. Intraoperative abdominal sonography was done for the appropriate evaluation of the place of resection (Figure 4 and Figure 5). Combined surgery allowed abdominal cavity inspection and spontaneous drainage of hematosalpinx and hematometra was observed (Figure 6, Figure 7, Figure 8, Figure 9 and Figure 10). An intravaginal catheter was placed for seven days to prevent early vaginal stenosis.

### 2.4. Follow-Up

The patient came for a health check after one week and was followed up for prevention of complications like vaginal stenosis and recurrence of obstruction. Hormonal therapy with combined estrogen–progesterone pills was administered to avoid heavy periods and possible inflammation which could accelerate the stenosis process. At 6 months follow up, the patient was free from vaginal stenosis, rUTI and recurrence of obstruction. We obtained written parental permission to present this case study.

Table 1 presents a management flow chart of patients with obstructed hemivagina with ipsilateral renal agenesis (OHVIRA).

## 3. Discussion

OHVIRA syndrome is a rare congenital urogenital anomaly derived from Mullerian tract abnormality [16]. The characteristic triad of this syndrome is didelphys uterus, obstructed hemivagina and ipsilateral renal agenesis [16]. OHVIRA often is accompanied by other anatomical disorders like a longitudinal or transverse vaginal septum [17]. Also, renal agenesis, urinary retention or unexplained recurrent UTIs should always catch the surgeon’s attention for possible congenital anomalies. However, other accompanying renal anomalies, such as renal dysplasia, a double collecting system and ectopic ureter are possible; although, only a limited number of such cases have been reported [18,19]. Symptoms like acute abdominal pain, dysmenorrhea and urinary disorders can mislead clinicians to make an inaccurate diagnosis. 

This case highlights the potential pitfalls and challenges in gynecologic surgery in adolescence. It also emphasizes that uterus didelphys with obstructed hemivagina (OHVIRA) should be considered in the differential diagnosis of adolescent girls presenting with acute abdominal pain, recurrent UTI and CAKUT [20,21]. We also aimed to highlight the crucial role of a detailed diagnostic process and correct management for future family planning in OHVIRA patients. 

Sometimes, a condition misinterpreted as dysmenorrhea and normal menstrual blood flow through the unobstructed hemivagina can cause delay in reaching a correct diagnosis. Dysmenorrhea is reported by 30–90% of young female patients and “irregular menstrual bleeding” in up to 43% [22]. The presence of menarche does not mean that an anatomical problem does not exist. A comprehensive interview with regard to medical history is an important part of the diagnostic process. In the meantime, the first menstruation in our patient came from a healthy uterus, which misled the clinician to make a good diagnosis and excluded the anatomical cause of the symptoms.

Pelvic pain in adolescents is often a puzzle, and suspicion and knowledge about different causes are important for accurate diagnosis. As urogenital abnormalities are rare, they can be mistaken for ovarian cysts [23]. In our case, the second uterus was taken as an ovarian cyst, which prolonged the diagnosis. 

Recurrent UTI can be associated with specific women’s anomalies, but if not treated properly it can lead to upper urinary tract infections like glomerulo- or pyelonephritis [2,3,4,24,25]. Therefore, urinary tract disorders are usually the first sign of unrecognized urogenital tract disorders. 

Often forgotten, endometriosis in adolescence can provoke dysmenorrhea, chronic pelvic pain, and, in consequence, develop endometrial cysts, which could imitate large abdominopelvic masses [26].

Such abnormalities like vaginal septum, urogenital sinus, rudimentary horn, and others should be in mind when counselling a female adolescent with nonspecific symptoms. The need for precise follow up is necessary in patients with CAKUT, and it must be noted that renal agenesis and other things could be associated with congenital anomalies of female reproductive organs [2].

MRI imaging is the gold standard for diagnosis with higher sensitivity in detecting the uterine morphology and the continuity of the vagina when compared to ultrasonography [6]. However, high cost and sometimes a long waiting time for the result, constitutes the limits of this investigation. In this case, abdominal sonography, which was thought to be suitable for virgins, misled the radiologist and the congenital abnormality was mistaken for an ovarian cyst. Urogenital anomalies may be overlooked, because of the rare prevalence of these conditions. Transrectal sonography is feasible when a transvaginal method cannot be performed; though it can be helpful, it is rarely accepted by young patients [26]. 

In the paediatric and adolescent population, scan techniques are essentially limited to a transabdominal or transperineal approach. Therefore, transperineal scanning could reveal a distended vagina and/or uterus filled with fluid containing low-level echoes [27]. Furthermore, in the evaluation of vaginal pathologies, the use of three-dimensional/four-dimensional ultrasound should be considered, given its high sensitivity and specificity [28]. 

Gynecologic surgeons face many challenges when considering complex gynecologic surgery, especially in children. Anticipating the intraoperative risks and a careful step by step, surgical plan should be an integral part of each procedure in children with congenital anomalies.

To date, several surgical techniques for OHVIRA management have been reported, such as puncture of the vaginal wall, single-stage vaginoplasty, mini-laparotomy, even unilateral hysterectomy and using a vaginal mold or stent [8,29]. Septum and vaginal trauma, which often occur because of using retractors, lead to searching for new methods that will allow them to reduce such complications. According to data, a new “No-touch” technique is safe and effective, provides good visualization and helps to protect the septum from trauma. Authors also reduce lower risk of vaginal stenosis, adhesions and postoperative pain [29]. Grünberger described a modification of the surgical approach, which is called Z-plasty [30]. Van Bijsterveldt et al. proposed a combined abdominal–vaginal method that can be used in patients with a higher risk of restenosis after surgery. Layperson modified previous author’s technique using a balloon catheter to provide traction for the vagina, which prevented vagina stenosis [31].

It is technically difficult to puncture the thick fibrotic tissue of the hemivaginal septum with a needle. Due to such a proceeding, we expose the patient to unnecessary interventions, like minilaparotomy. Therefore, we offered our patient intraoperative abdominal sonography to directly evaluate the accurate and safe place of puncture and septal resection.

Minimally invasive surgery has acquired more and more space in the surgical panorama. Furthermore, it has many advantages in adolescent gynecology. Some of the authors discussed the role of laparoscopy in the management of OHVIRA [18,32,33]. In our opinion, a concomitant laparoscopic approach allows us to particularly evaluate the smaller pelvis.

Hydrometrocopes and those associated with OHVIRA recurrent blood flow can develop hematosalpinx and, in consequence, endometriosis [34]. During laparoscopy we observed the drainage of hematosalpinx; therefore, the efficacy of our treatment was ‘under eye’ control. We have also found active endometriosis lesions, which confirmed that late recognition of OHVIRA can have serious consequences.

The necessity of the unilateral hysterectomy of one uterus is questionable. First of all, such a procedure is not a simple one for children. Another point is regarding the future family planning in patients with congenital anomalies.

In contrast to other authors, we believe that concomitant laparoscopic surgery is necessary in the surgical approach for OHVIRA patients [18,32], not only for clarification of the diagnosis, but also for the evaluation of pelvis anatomy and the potential consequences of unrecognized diseases like hematosalpinx and endometriosis. Perhaps, preoperative evolution of the Ca-125 marker indicates a possible development of early endometriosis. Furthermore, laparoscopic surgery allows intraoperative observation of drainage from the uterus and the efficacy of our treatment.

Because of the higher risk of vaginal restenosis in patients with OHVIRA syndrome, there is a strong need for long-distance follow up in such patients. Sometimes surgical resection is necessary. Early postoperative management with antibiotics and oral contraceptives is necessary to avoid vaginal infection and future restenosis.

Follow up of OHVIRA requires prolonged monitoring of solitary kidney function and guidance enabling satisfactory sexual intercourse and delivery. Girls with renal agenesis should be carefully monitored for possible genital tract anomalies, and, conversely, girls with vaginal or uterine anomalies should be monitored for renal malformations [35].

This study has some limitations. First, most reports related to OHVIRA comprise only individual cases, which are caused by the rarity of this disorder. Only some studies showed a larger number of cases. Also, some data from medical history was missing or the value of medical images was poor.

The purpose of this study was to outline the correct management pathway and surgical approach concerning OHVIRA, which could vary not only due to anatomical differences, but also due to medical advances and the possibility of delayed diagnosis.

## 4. Conclusions

OHVIRA conditions may be associated with rUTI and CAKUT. Our findings show, that a careful evaluation of the urinary tract should be recommended in all OHVIRA patients.

However, delayed diagnosis may result in late complications, including retrograde tubal reflux and endometriosis. Unrecognized and improperly treated congenital malformations of the vagina, cervix, and uterus, although rare, may have severe implications for future family planning in OHVIRA patients.

In our case, transperineal sonography turned out to be a helpful tool in the early diagnosis of OHVIRA. Combined management of laparoscopy and vaginoscopy should be considered in all female patients with suspicion of congenital abnormalities and acute abdominal symptoms as standard procedure.

Vaginal septum resection with drainage seems to be the ideal surgery to restore normal vaginal function. After surgery, adolescent patients should undergo regular follow up visits in order to prevent an adhesion-formation recurrence after vaginal septum resection.

## Figures and Tables

**Table 1:** Management flow chart of patients with obstructed hemivagina with ipsilateral renal agenesis (OHVIRA). Flowchart abbreviations: UTI—urinary tract infection; US—ultrasound.

Diagnostic and Treatment Flow-Chart of OHVIRA Syndrome
Step 1. Preoperative management Patients age (mostly occurs in young females), accurate history: time of first period, gradually increased symptoms, the appearance of symptoms during menarche, symptoms increase with each subsequent period, recurrent UTI, urinary disorders [8,9]. Perform physical examination. Next to the transabdominal US, transperineal and transrectal US can be useful in emergency situations to accurately access the place of abnormality [10]. MRI imaging should be considered as the “gold standard in the diagnostic process” [11]. Planning of the surgery and step by step proceedings is important while operating on patients with urogenital abnormalities. An interdisciplinary collaboration of a urologist and gynecologist is often necessary to treat these patients correctly.
Step 2. Preoperative management Preoperative counselling with patients, often with psychological assist is essential to support families. The information about future family planning and chanced of spontaneous pregnancy should be given precisely before the surgery [12]. However, hormonal therapy with continuous oral contraceptives should be considered in pre-operative management to give a time for preoperative preparation [13].
Step 3. Surgical management Surgery is necessary when acute abdominal symptoms are present. “Wait and see” approach is only possible when the clinical situation allows it. Perform laparoscopy and vaginoscopy in order to achieve the correct diagnosis and treat concomitant hematosalpinx and endometriosis [14]. Intraoperative US is helpful to evaluate the place of resection. Unnecessary lengthening the time to diagnosis, contributes to unindentent consequence.
Step 4. Postoperative management The insertion of uterine catheter filled with saline into a place of stenosis allow to avoid the risk of possible restenosis [15]. Continuous oral contraceptives are recommended to avoid possible consequences like the risk of restenosis.
Step 5. Postoperative management Pharmacological treatment with non-steroidal anti-inflammatory drugs (NSAIDS) should be avoided as they can cause the damage of renal structure [12].
Step 6. Follow-up At 6–12 month after the surgery, urologist re-counselling should be made to decide if the patient requires other imaging tests like uro-scintigraphy to evaluate the function of heathy kidney [2].

## Data Availability

The authors confirm that the data supporting the findings of this study are available within the article.
